# Separation and Purification of Two Saponins from *Paris polyphylla* var. *yunnanensis* by a Macroporous Resin

**DOI:** 10.3390/molecules27196626

**Published:** 2022-10-06

**Authors:** Xiaoya Zhang, Junli Wu, Long Qin, Guangxi Wang, Ping Li, Anmin Yu, Aizhong Liu, Rui Sun

**Affiliations:** Key Laboratory for Forest Resources Conservation and Utilization in the Southwest Mountains of China, Ministry of Education, College of Forestry, Southwest Forestry University, Kunming 650224, China

**Keywords:** macroporous resins, separation, saponins, *Paris* *polyphylla* var. *yunnanensis*

## Abstract

An effective method for separating and purifying critical saponins (polyphyllin II and polyphyllin VII) from a *Paris polyphylla* var. *yunnanensis* extract was developed in this study which was environmentally friendly and economical. Static adsorption kinetics, thermodynamics, and the dynamic adsorption-desorption of macroporous resins were investigated, and then the conditions of purification and separation were optimized by fitting with an adsorption thermodynamics equation and a kinetic equation. Effective NKA-9 resin from seven macroporous resins was screened out to separate and purify the two saponins. The static adsorption and dynamic adsorption were chemical and physical adsorption dual-processes on the NKA-9 resin. Under the optimum parameters, the contents of polyphyllin II and polyphyllin VII in the product were 17.3-fold and 28.6-fold those in plant extracts, respectively. The total yields of the two saponins were 93.16%. This research thus provides a theoretical foundation for the large-scale industrial production of the natural drugs polyphyllin II and polyphyllin VII.

## 1. Introduction

Due to their novel structures, high curative effects and fewer adverse reactions of the active components of natural drugs, especially considering special physiological activities, many drugs with unique therapeutic effects have been developed recently. However, many active natural components with complex structures are difficult to obtain when their content is low in given tissues. Therefore, the development and utilization of separation and purification technologies are particularly important to enrich the sources of active natural products. Conventional separation and purification methods, such as silica gel column chromatography, high-speed counter current chromatography and liquid–liquid extraction, are widely applied to the separation of various kinds of natural products, but these methods often have several limitations, e.g., environmental pollution, toxicity, and high cost [1,2,3]. In recent years, with the progress of separation and analysis technology, adsorption-desorption processes by macroporous resins have been developed and widely used to purify natural product extracts because they are easily scalable, inexpensive, reusable, non-toxic, and do not cause secondary pollution [4,5,6]. Macroporous resins have been used for separating and purifying diverse natural products such as phenols, flavonoids, glycosides and saponins [7,8,9,10,11,12]. Cheng et al. (2021) used XAD7HP resin to isolate and purify polyphenols extracted from areca seeds, resulting in the purity of the polyphenol increasing from 44.03% to 85.96%. Jiang et al. (2020) studied the separation and purification process of chlorogenic acid from *Eucommia ulmoides* Oliv. With NKA-II resin and found that the purity of chlorogenic acid from the extract increased 6.21-fold from 1.9% to 11.8%, and the recovery rate reached 87.9%.

**Figure 1:**
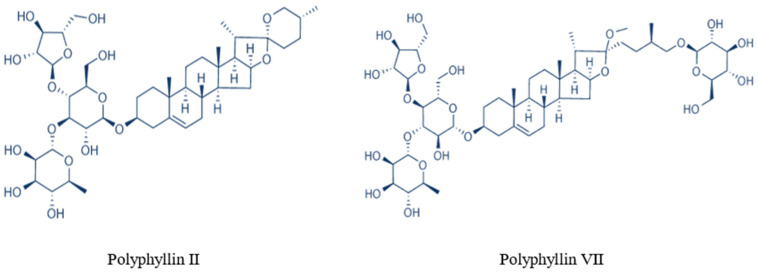
Chemical structures of two saponins.

Paridis Rhizoma, a traditional medicine in China, is prepared using the dry rhizome of *Paris polyphylla* var. *yunnanensis*, which is mainly distributed in southwest China (such as in the Yunnan, Guizhou, and Sichuan Provinces). The main medical benefits of Paridis Rhizoma include detoxification, reduction of swelling and pain, and it is often used to treat sores, carbuncles, sore throat, snake and insect bites in traditional Chinese medicine [13]. In the cosmetic industry, it was used for treating acne, repairing skin, and its anti-inflammatory and antibacterial characteristics. Studies have found that the main medical benefits of Paridis Rhizoma are due to the medical functions of diverse polyphyllins (compounds of plant polyphenols) such as polyphyllins I, II, III, IV, V, VI and VII [14,15,16]. Although the separation, purification, and medical functions of polyphyllins have been widely studied in Paridis Rhizoma, studies investigating the separation and purification of single compounds remain scarce. The structure of polyphyllin II and polyphyllin VII (Figure 1) from *P*. *polyphylla* is complex. Polyphyllin II and polyphyllin VII have reported that they have anticancer, anti-inflammatory, and antioxidant activity [17,18]. For polyphyllin II, several studies have demonstrated that it can physiologically induce apoptosis and actively treat lung cancer as an adjuvant drug [19,20,21]. For polyphyllin VII, studies have found that it can inhibit the growth, invasion and migration of tumor cells and induce tumor cell apoptosis through different pathways and mechanisms [22,23]. Therefore, in order to obtain natural drugs polyphyllin II and polyphyllin VII, separation and purification using macroporous resins is a good approach. There are many kinds of macroporous resins, and different formations of substances are compatible with different resins. According to previous studies, the particle size, pore size and polarity of adsorption particles can all affect the adsorption process [7,11]. The properties of functional groups on the surface of adsorption particles, including polarity, acid-base and hydrogen bonding ability, affect the adsorption selectivity and adsorption behavior [24,25]. Generally, adsorption kinetics and thermodynamic fitting are used to understand and estimate adsorption behavior and adsorption ability of resin [26,27,28]. To date, HPD-600 resin screened from seven resins (HPD-400, HPD-600, HPD-826, X-5, DM-130, XAD-2, ADS-17, and AB-8) has been used to isolate and prepare polyphyllin II and dioscin from the stems and leaves of *P. polyphylla*. However, polyphyllin VII has not been studied, and adsorption kinetics and thermodynamic fitting analyses have not been carried out [29].

Therefore, polyphyllin II and polyphyllin VII from the whole plant of *P. polyphylla* var. *yunnanensis* were separated and purified by macroporous resins. Based on the adsorption-desorption behaviors, kinetics and thermodynamics, suitable resins for these two compounds were identified. The most suitable resin also exhibited dynamic adsorption, which is valuable for the purification process. This study is the first to optimize the purification process of polyphyllin II and polyphyllin VII using static and dynamic adsorption kinetics from two perspectives. The objective of this research is to develop an effective method for separating and purifying critical saponins (polyphyllin II and polyphyllin VII) from a *P. polyphylla* var. *yunnanensis* extract. In the present work, we used extracts from the whole plant and used various resins, resulting in a theoretical foundation for the large-scale industrial production of the natural drugs polyphyllin II and polyphyllin VII.

## 2. Materials and Methods

### 2.1. Reagent and Materials

The standards of polyphyllin II and polyphyllin VII with 98% purity were purchased from Shanghai NatureStandard Bio-Technology Co., Ltd. (Shanghai, China). Acetonitrile (J&K Scientific Ltd., Beijing, China) was used for high performance liquid chromatography (HPLC) analysis. Analytical grade ethanol and methanol were acquired from Guangdong Guanghua Sci-Tech Co., Ltd. (Guangdong, China). Ultrapure water was used in all experiments from ELGA (PP010XXM1, UK). All solvents prepared for HPLC were filtered through a syringe filter (0.22 µm pore size) before use. D101, HPD-500, XAD7HP and AB-8 resins were purchased from Beijing Solarbio Science Technology Co., ltd. (Beijing, China). HPD-100, NKA-9 and HP-20 resins were purchased from Shanghai Yuanye bio-Technology Co., Ltd. (Shanghai, China). Macroporous resins were washed with 95% (*v*/*v*) ethanol, soaked for 24 h at room temperature, and then removed with distilled water. Since polarity and the physical properties (such as size of surface area, pore diameter and moisture content) of different resins can often affect the adsorption and desorption of compounds [30], different resins including non-polar resins (HPD-100, D101 and HP-20), weak polar resins (XAD7HP and AB-8) and polar resins (NKA-9 and HPD-500) with different physical properties were used in this study (Table 1).

### 2.2. Preparation of Sample Solution

**Figure 2:**
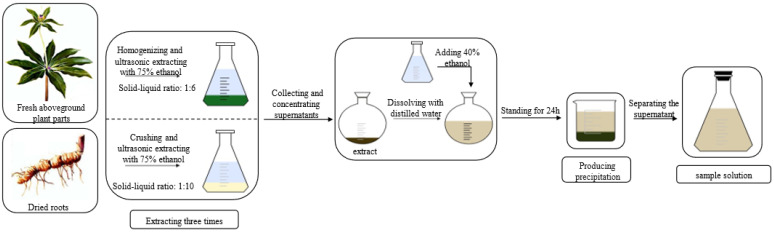
Experimental scheme for extraction of polyphyllin II and polyphyllin VII.

Seedings of *P. polyphylla* var. *yunnanensis* were from the plantation base of the Kunming Jiange Traditional Chinese Medicine Planting Co., Ltd. (Yunnan, China) with a voucher specimen stored in KUN (Paris-01). These seedlings were cultivated without the application of fertilizers under the forest in a natural environment (Latitude: 102.4564° N, Longitude: 25.1516° E). The preparation process of sample solution is shown in Figure 2. In total, 50 plants (7 to 8-year-old) were sampled. Fresh roots (3.93 kg) were dried using the thermostatic blast drying oven (DHG-9145A, Shanghai Yiheng Technology Co., Ltd., Shanghai, China) at 50 °C for 5 days. Dried roots (2.55 kg) were crushed and extracted with 75% ethanol at 50 °C for 30 min in an ultrasonic bath (SK5210HP, Shanghai Kudos Ultrasonic Instrument Co., Ltd., Shanghai, China), and the process was repeated three times. The solid-liquid ratio was 1:10. Since increasing evidence was showing that the fresh aboveground parts (including stem and leaf) often also contain active polyphyllin compounds [31,32], we pooled the dried root and fresh aboveground tissues to extract. Fresh aboveground parts (0.65 kg) were homogenized and extracted with the same method. The solid-liquid ratio was 1:6. The collected supernatants of all extracts were combined and concentrated to dryness at 50 °C in a rotary evaporator device (N-1300, Shanghai Ailang Instrument Co., Ltd., Shanghai, China). The residue (238.49 g) was dissolved with distilled water and the same volume of 40% ethanol was added. A dark deposit appeared. The supernatant and deposition were separated by centrifuge (5805CJ064433, Eppendorf, German) at 12,000 rpm/min for 10 min. The supernatant was prepared as sample solution. The concentrations of polyphyllin II and polyphyllin VII were 2.391 mg/mL and 2.035 mg/mL in the sample solutions. The contents of polyphyllin II and polyphyllin VII in the sample solution were 2.04% and 1.74%, respectively.

### 2.3. HPLC Analysis of Polyphyllin II and Polyphyllin VII

**Figure 3:**
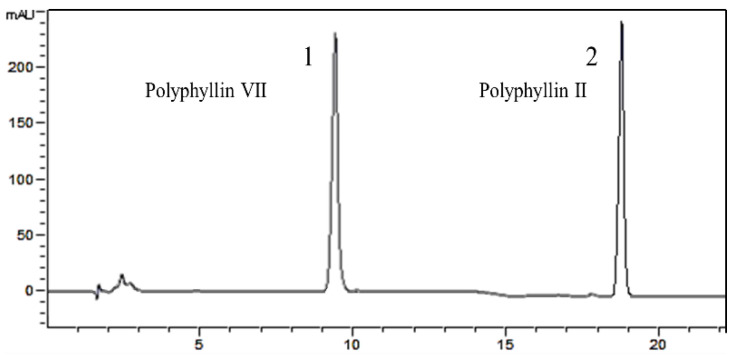
HPLC chromatograms of standards: polyphyllin VII (1) and polyphyllin II (2).

Polyphyllin II and polyphyllin VII were analyzed by an Agilent 1260 liquid chromatography system (Agilent Technologies, Santa Clara, CA, USA) equipped with a quaternary pump, a manual injector, a DVD ultraviolet detector, and an EC-C_18_ column (250 mm × 4.6 mm id; 4 µm). The mobile phase consisted of A (water) and B (acetonitrile) using the gradient elution method (Table 2) at a flow rate of 1 mL/min. The UV detection wavelength was set at 203 nm, the column temperature was maintained at 30 °C, and the injection volume was 10 µL. The chromatogram for standards is shown in Figure 3. The retention time of polyphyllin II and polyphyllin VII were 20.070 min and 9.534 min, respectively. Quantitative analysis was measured by the external standard method, which showed a good linearity over the range of 0.016–1 mg/mL. The standard curse for polyphyllin II is y = 2875.3x + 12.659 (R^2^ = 0.9993), and for polyphyllin VII it is y = 3107.6x − 3.207 (R^2^ = 0.9970), where y is the peak area of polyphyllin II and polyphyllin VII, and x is the concentration (mg/mL).

### 2.4. Optimization of Resins

The static adsorption and desorption tests were accomplished as follows: 9 mL of sample solution prepared as described in Section 2.2 was added into test tubes containing 0.2 g (dry weight) of seven pretreated types of resins (XAD7HP, HPD-100, HPD-500, NKA-9, D101, AB-8 and HP-20). Next, test tubes were kept in an incubator shaker (120 rpm/min) at 25 °C for 24 h. The resins were separated from the solution and then adequately washed with deionized water. The adsorbed resins were desorbed with 9 mL 75% (*v*/*v*) ethanol solution in the same condition with the adsorption.

The following formulas were used to calculate and evaluate the adsorption properties.

(1) $$Q_{e}=\frac{V_{1}\left(C_{0}-{\;C}_{e}\right)}{m}$$

(2) $$A=\frac{\left(C_{0\;}-{\;C}_{e}\right)}{C_{0}}\;\times \;100\%$$

where Q_e_ represents the adsorption capacity at adsorption equilibrium (mg/g resin); A represents the adsorption ratio (%), which is the percent of the adsorbed quantity to the initial quantity after equilibrium; C_0_ and C_e_ are the initial and equilibrium concentrations of polyphyllin II and polyphyllin VII, respectively; V_1_ is the volume of the sample solution (mL); and m is the weight of the resins added (g, dry weight).

The following formula was used to calculate and evaluate the desorption property.

(3) $$D=\frac{V_{2}C_{1}}{V_{1}\left(C_{0}-{\;C}_{e}\right)}\;\times \;100\%$$

where D represents the desorption ratio (%); C_1_ is the concentration of the solution after desorption; V_2_ is the volume of desorption solution; and C_0_, C_e_ and V_1_ are the same as mentioned above.

### 2.5. Static Adsorption Kinetic and Fitting Equations

5 mL sample solutions were added into test tubes with 0.1 g (dry weight) of three ideal resins selected in Section 2.4. The mixtures were shaken at 25 °C in an incubator shaker (120 rpm/min). The time was set at 1, 2, 4, 8, 20, 40, 60 and 120 min. The kinetic curves of three resins were prepared to select the optimum resin, i.e., the curves of the adsorption quantity as a function of time at a constant temperature. The results were fitted to a pseudo-first-order model (4), a pseudo-second-order model (5), a Weber-Morris intragranular diffusion kinetic model (6), and a Bangham model (7) for analyzing the adsorption behavior of the resins.

Pseudo-first-order model equation:

(4) $$\log\left(Q_{e}-Q_{t}\right){=\mathrm{logQ}}_{e}-\frac{K_{1}t}{2.303}$$

Pseudo-second-order model equation:

(5) $$\frac{t}{Q_{t}}=\frac{1}{K_{2}Q_{e}^{2}}+\frac{t}{Q_{e}}$$

Weber-Morris intragranular diffusion model equation:

(6) $${\;Q}_{t}{=K}_{p}t^{0.5}$$

Bangham model equation:

(7) $$\mathrm{loglog}\left(\frac{C_{0}}{C_{0}-Q_{t}m}\right)=\log\left(\frac{\mathrm{mK}}{2.303V_{1}}\right)+\mathrm{alogt}$$

where K, K_1_, K_2_, and K_p_ are the adsorption rate constants under their respective models; Q_e_ and Q_t_ represent the adsorption quantities of compounds at equilibrium and at time t, respectively; and a is the correlation coefficient of the Bangham model [33,34,35,36].

### 2.6. Isothermal Curve and Adsorption Thermodynamics Equation

Adsorption isotherm experiments were carried out by bringing seven aliquots of sample solutions at different concentrations into contact. Various volumes of 20% (*v/v*) ethanol solution were added to the initial sample solution to obtain different concentrations of polyphyllin II and polyphyllin VII in samples. A nine mL sample solution was added into each test tube with 0.2 g (dry weight) of the optimum resin obtained from the above experiment and was shaken at 25, 30 and 35 °C for 120 min, respectively. Finally, the sample solutions were made with different concentrations of polyphyllin II (0.349, 0.817, 1.178, 1.681, 2.287, 2.521, and 3.698 mg/mL), and polyphyllin VII (0.247, 0.517, 0.699, 0.911, 1.196, 1.364, and 1.891 mg/mL). Fitting the data with different isotherm models is important for delineating the adsorbent–adsorbate interactions and the design of the adsorption process. Langmuir and Freundlich isotherm models are widely used.

The Langmuir isotherm model equation is usually given as:

(8) $$Q_{e}=\frac{Q_{m}K_{L}C_{e}}{{1+K}_{L}C_{e}}$$

The Freundlich isotherm model equation is expressed as:

(9) $${\mathrm{lnQ}}_{e}{=\mathrm{lnK}}_{F}+\frac{1}{n}{\mathrm{lnC}}_{e}$$

where Q_m_ indicates saturated adsorption capacity (mg/g); K_L_ represents adsorption energy correlation constant; K_F_ is the Freundlich adsorption capacity coefficient; and 1/n is the correlation coefficient of resin capacity [37,38].

The Thermodynamics parameters, including enthalpy, entropy and Gibbs free energy were calculated using the van’t Hoff dependency:

(10) $$\frac{{d\;\mathrm{lnK}}_{a}}{d\;T}=\frac{\Delta H{}^{\circ}}{{\mathrm{RT}}^{2}}$$

and from the following relationships:

(11) ΔG°=−nRT

and

(12) $${\mathrm{lnK}}_{a}=\frac{\Delta S{}^{\circ}}{R}-\frac{\Delta H{}^{\circ}}{\mathrm{RT}}$$

where ∆G°, ∆S°, and ∆H° are Gibbs free energy, entropy of adsorption, and enthalpy of adsorption (kJ/mol); R is the universal gas constant; T is the absolute temperature (K). K_F_ is the Freundlich adsorption capacity coefficient; and n is the correlation coefficient of resin capacity [39,40,41].

### 2.7. Dynamic Adsorption-Desorption Behavior on Resin Column

Dynamic adsorption-desorption tests were carried out in a glass column (750 mm × 12 mm id) wet-packed with 4 g (dry weight) selected resin. The bed volume (BV) of the resin was 5.5 mL, and the resin bed length was 7 cm. The sample solution with 3.413 mg/mL polyphyllin II, and 2.323 mg/mL polyphyllin VII was loaded onto the column. The leak flow rate was 1 mL/min. One mL effluent was collected at 3 mL intervals. After loading, the adsorbed column was washed with deionized water and was then desorbed with 60% ethanol. One mL eluent was collected at 1–3 mL intervals.

Finally, the optimum sample loading and elution volumes were obtained by the above steps. The sample solution was loaded on the resin column under the same conditions again. The column was washed with distilled water at first, and then was desorbed with 16 BV different concentrations of ethanol solutions (20%, 30%, 40%, 50%, 60%, 70% and 80%). The flow rate was 1 mL/min, and polyphyllin II and polyphyllin VII in each eluent were analyzed by HPLC.

### 2.8. Dynamic Data Fitting Adsorption Kinetic Equation

The data from the dynamic leakage curve in Section 2.7 can be used to fit the adsorption kinetic equation and study the adsorption reaction on the resin column. A pseudo-first-order model, pseudo-second-order model, and Weber-Morris intragranular diffusion kinetic model were also used for analyzing the adsorption behavior of the resins. The equation information is contained in Section 2.5.

### 2.9. Statistical Analysis

The data fitting and analysis were carried out using Excel (Microsoft Office) and Origin Pro 8.0 (Origin Lab Corporation, Northampton, MA, USA). Figures were drawn using Origin Pro 8.0. The mean and standard deviation of three replicate experiments were reported.

## 3. Results and Discussion

### 3.1. Static Adsorption and Desorption

**Figure 4:**
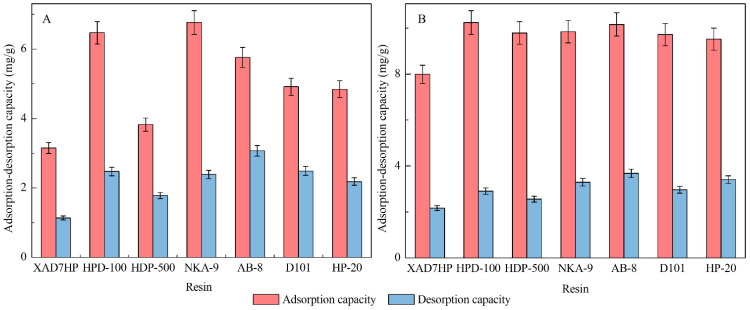
Static adsorption-desorption of polyphyllin II (**A**) and polyphyllin VII (**B**) on seven resins.

The static adsorption-desorption of polyphyllin II and polyphyllin VII on different resins was investigated. The result is shown in Figure 4. For polyphyllin II, the adsorption and desorption capacities of NKA-9, HPD-100, and AB-8 were obviously better than those of the other resins. For polyphyllin VII, higher adsorption and desorption capacities of AB-8, HPD-100, NKA-9, D101, and HP-20 were found. HPD-100, D101, and HP-20 are nonpolar; NKA-9 is polar; and AB-8 is weakly polar (Table 1). According to the principle of ‘similar dissolution’, polar substances are easily adsorbed and desorbed by polar resins. In addition, because the adsorption action occurred through van der Waals forces or hydrogen bonding, the chemical effect was reversely easy to desorb [42]. Therefore, NKA-9 and AB-8 had excellent adsorption-desorption properties for the two saponins due to their similar characteristics. It has been reported that other physical parameters of resins, including surface area and average pore diameter, are closely related to their adsorption capacity, e.g., for HPD-100, D101, and HP-20. The larger the specific surface area of the resin and the more appropriate the action aperture per unit mass, the larger the number of effective components that can be adsorbed on the surface. The surface area of HPD-100 is approximately 650–700 m^2^/g (Table 1), making it the largest of all the resins, which might explain why it is more suitable than D101 and HP-20. Thus, we screened out NKA-9, AB-8, and HPD-100 for subsequent investigation. The static adsorption and desorption were usually used to estimate suitable resins. Similarly, Yang et al. screened out suitable HPD-100 resin from six resins when purifying the flavonoids from the flower of *Abelmoschus manihot* (L.) Medic [43]. Generally, the static adsorption and desorption stage is necessary for the initial screen of suitable resins to isolate and purify a given compound.

### 3.2. Adsorption Kinetic Curves and Equations

**Figure 5:**
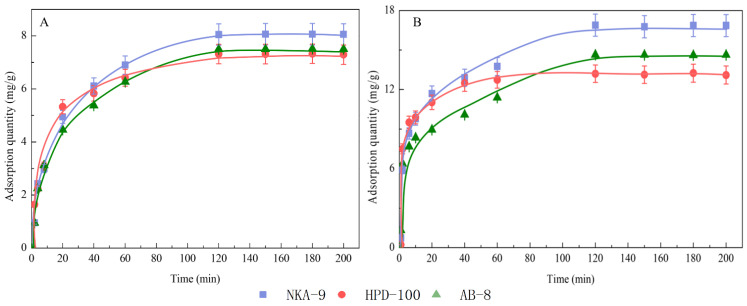
Static adsorption kinetics curve of polyphyllin II (**A**) and polyphyllin VII (**B**) on NKA-9, HPD-100, and AB-8 resins.

**Figure 6:**
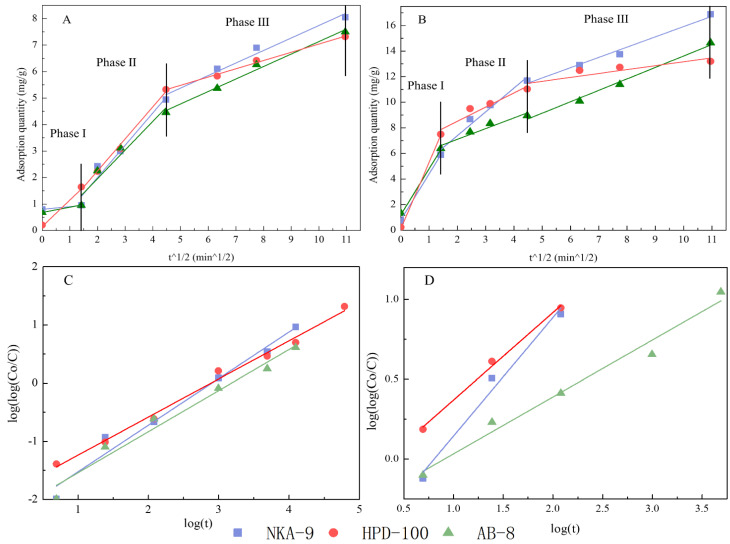
Weber-Morris intragranular diffusion kinetic model (**A**,**B**) and Bangham model (**C**,**D**) of polyphyllin II (**A**,**C**) and polyphyllin VII (**B**,**D**) on NKA-9, HPD-100, and AB-8.

The adsorption procedure of NKA-9, AB-8, and HPD-100 resins in different periods under static conditions is shown in Figure 5. For polyphyllin II and polyphyllin VII, the trends of the three resins were consistent. Clearly, the adsorption capacity increases with time. The adsorption rate was fast in the first 20 min; from 20 to 60 min, the adsorption rate gradually decreased; and finally, the adsorption approached equilibrium and stability at 120 min. To further understand the resin behavior, pseudo-first-order, and pseudo-second-order model-fitting parameters were determined, as shown in Table 3. The Weber-Morris intragranular diffusion kinetic model and Bangham model fitting images are shown in Figure 6.

Generally, the pseudo-first-order model is used to describe the equilibrium adsorption capacity and the rate of solute adsorption by an adsorbent. The pseudo-second-order model indicates that the main factors affecting adsorption (such as polarity) are related to chemical adsorption. The more consistent the data are with the model, the more chemical adsorption contributes to the adsorption process [44,45]. As shown in Table 3, a correlation was obtained in the pseudo-first-order model (R^2^ ≥ 0.95) for polyphyllin II, so the model can be used to describe the adsorption behavior and the fitted equilibrium adsorption capacities on the three resins. The Qe values of the NKA-9, HPD-100 and AB-8 resins were 7.925, 6.618 and 6.64, respectively. Clearly, the Qe value of NKA-9 resin was the largest. The pseudo-second-order model had a high correlation on the NKA-9 and AB-8 resins (R^2^ ≥ 0.95), but the R^2^ value for the HPD-100 resin was less than 0.95. Thus, the pseudo-second-order model could describe the adsorption process of only the NKA-9 and AB-8 resins. The polar NKA-9 and AB-8 resins were mainly controlled by chemisorption, consistent with the structure of two resins with a hydrophilic polar group attached to the framework of cross-linked styrene-divinylbenzene copolymer. The nonpolar HPD-100 resin with styrene structure was mainly affected by physical adsorption. For polyphyllin VII, the pseudo-first-order model had a low correlation (R^2^ ≤ 0.95), which means that it is not suitable for simulating the adsorption of this substance. The reason for this finding might be that the pseudo-first-order kinetic model is more suitable to represent the initial process of adsorption. The pseudo-second-order model had a high correlation with the NKA-9 resin (R^2^ ≥ 0.95), but the AB-8 and HPD-100 resins had the opposite correlation (R^2^ ≤ 0.95). This means that NKA-9 resin-adsorbed polyphyllin VII is mainly affected by chemical adsorption, which is consistent with the result of polyphyllin II. The weakly polar AB-8 resin and nonpolar HPD-100 resin adsorbed polyphyllin VII by physical adsorption.

The Bangham model can be used to check whether pore diffusion was the only rate-controlling step or not in the adsorption system [35]. The Bangham model fitting images of polyphyllin II and polyphyllin VII on NKA-9, HPD-100, and AB-8 resins are shown in Figure 6C,D. In the Bangham model equation, a is the correlation coefficient. When a value is close to 0.5, the diffusion is pure pore diffusion, while, when a value is close to 1.0, the diffusion is multiple actions. Thus, according to the a values shown in Table 3, the adsorption of these two saponins on HPD-100 resin is mainly pore diffusion (a = 0.655/0.548), and the adsorption of polyphyllin II on NKA-9 resin is affected by multiple adsorption factors (a = 0.801). The Weber-Morris intragranular diffusion model is usually used to describe three phases of adsorption: preadsorption (Phase I), main adsorption (Phase II), and the equilibrium procedure (Phase III). The main effects on the resins are liquid film diffusion, particle diffusion and equilibrium diffusion [42,46,47]. Three phases exhibiting linear relation were obtained under the Weber-Morris model (Figure 6A,B). In phase II, the K_p_ value (representing the reaction rate) of NKA-9 was the largest among the three resins in Figure 6. It is possible that the NKA-9 resin functional group and the two saponins structures are compatible.

The loading capacity of the NKA-9 resin was greater than that of the other two resins according to the Q_e_ value of the pseudo-first-order model (Table 3). In the adsorption process of the pseudo-second-order model, the chemical adsorption controlled by the polarity and various interaction forces was more conducive to the subsequent polar elution, and the NKA-9 resin met the condition; as determined from the main adsorption process rate of the Weber-Morris intragranular diffusion model, the K_p_ value of the NKA-9 resin was higher (Table 3). Therefore, the NKA-9 resin was chosen as the most suitable resin for the next experiments. Comparing with those reported, we found that the pseudo-second-order model could be better to describe the resin adsorption process than the pseudo-first-order model; see Table 4 [48,49,50].

### 3.3. Adsorption Isotherms and Thermodynamics

**Figure 7:**
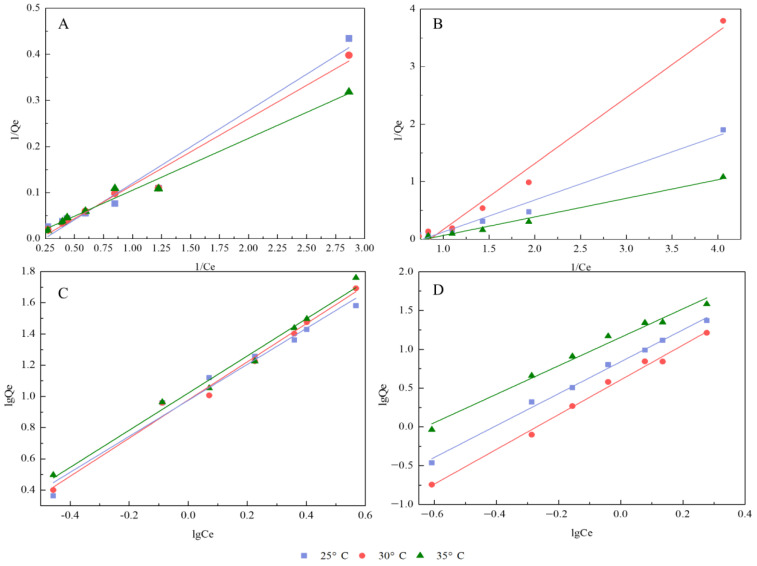
Linear correlation of polyphyllin II on NKA-9 at 25 °C, 30 °C, and 35 °C on the basis of the Langmuir (**A**) and Freundlich (**C**) models. Linear correlation of polyphyllin VII on NKA-9 at 25 °C, 30 °C, and 35 °C on the basis of the Langmuir (**B**) and Freundlich (**D**) models.

To characterize the adsorption behavior of polyphyllin II and polyphyllin VII on NKA-9, the effects of temperature on the adsorption were evaluated. The Langmuir and Freundlich models are frequently used theoretical models for understanding adsorption materials. The Langmuir equation describes homogeneous monolayer adsorption and predicts the maximum adsorption capacity. The Freundlich equation implies that the surface of the adsorbent is heterogeneous [49,51,52,53]. The corresponding parameters of the Langmuir and Freundlich thermodynamic equations at 25, 30 and 35 °C are summarized in Table 5, and Figure 7 intuitively reveals the linear correlations of the two saponins in the two models.

As shown in Table 5, high correlations were obtained with the Langmuir and Freundlich models with regard to describing the adsorption system (R^2^≥0.95). For polyphyllin II, the Q_m_ value (representing the saturated adsorption capacity) in the coefficient of the Langmuir equation increased with increasing temperature, which conforms to the law of chemisorption. The results were consistent with the results of pseudo-second-order dynamics. In the Freundlich model, the larger the K_F_ (representing the Freundlich adsorption capacity coefficient) and n (representing the correlation coefficient) values are, the better the adsorption performance [54]. The K_F_ and n values increased with temperature, which further demonstrated the best adsorption effect at 35 °C. For polyphyllin VII, it can be observed that the Q_m_ values were reduced from 25 °C to 30 °C, but the value at 35 °C was much higher than the first two. The adsorption process of polyphyllin VII experienced the dual effects of chemistry and physics. In the Freundlich isotherm model, the K_F_ and n values were also the largest at 35 °C.

The thermodynamic parameters enthalpy, entropy, and Gibbs free energy were calculated, and the results are shown in Table 6. The negative Gibbs free energy values indicated that the adsorption process was spontaneous in nature. The positive entropy values suggested that the adsorption was an entropy-driven process. In addition, the values of enthalpy were positive, demonstrating that the adsorption was a spontaneous and endothermic process [39].

This confirmed that the adsorption of the two saponins was mainly driven by a chemical process and was easily adsorbed by the NKA-9 resin. Among the three temperature conditions, the optimum temperature for the two saponins was 35 °C, which indicated that a higher temperature was beneficial to adsorption in a certain range. Indeed, the adsorption isotherm is critical in understanding energy conservation and the interaction between adsorbent and adsorbate, and many studies have investigated the adsorption isotherm performance of resins [30]. Previous studies investigated the adsorption isotherms of total flavonoids and three chromones on different resins, resulting in an optimum temperature of adsorption at 25 °C. Comparing these results, we found that the optimum adsorption temperatures of different resins were different, probably due to different resin polarity, molecular structures, and weights of the compounds (Table 7).

### 3.4. Dynamic Leakage Curve and Kinetic Fitting Equations

**Figure 8:**
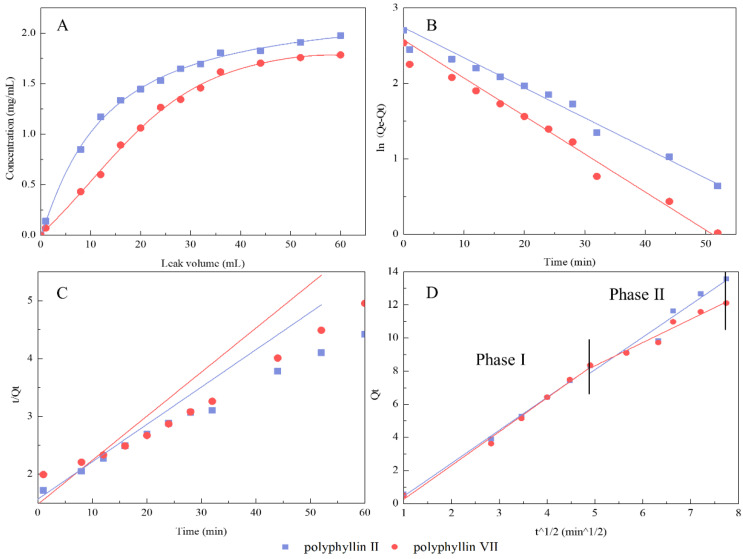
Linear correlation of leakage curve (**A**), pseudo-first-order model (**B**), pseudo-second-order model (**C**), and Weber-Morris intragranular diffusion model (**D**) of polyphyllin II and polyphyllin VII.

Polyphyllin II and polyphyllin VII in the sample solution were rapidly adsorbed by the resin at the initial 10 mL and slowly adsorbed from 10 to 40 mL until the resins in the column were close to saturation at 50 mL (Figure 8A). In contrast to that of polyphyllin II, the stationary equilibrium point of polyphyllin VII occurred earlier. If the volume of feed solution was selected according to polyphyllin VII, polyphyllin II would not reach adsorption saturation. By studying the adsorption behavior of the two saponins on a dynamic resin column, the volume of the sample solution can be further determined, and more information can be provided. Table 8 shows the kinetic equation parameters of dynamic adsorption, and more visual linearized forms are shown in Figure 8B–D.

For the two saponins, the pseudo-first-order model showed a significant linear correlation (R^2^ ≥ 0.97), as shown in Table 8. However, it did not show a correlation in the pseudo-second-order model, in which R^2^ was lower than 0.95. The dynamic resin column had a dual influence of physical and chemical actions. The Weber-Morris intragranular diffusion model revealed two-stage diffusion (Figure 8D). The preadsorption time was shortened, and the main adsorption time was increased. Three-stage adsorption was shortened to two-stage adsorption. The main adsorption was completed quickly, allowing us to appropriately adjust the amount of sample to achieve separation efficiency. According to the results above, the feed volume of the solution was chosen to be 55 mL (10 BV). To our knowledge, the kinetic behavior of dynamic adsorption on the resin column was described by the kinetic fitting equation for the first time in this study.

### 3.5. Optimization of Dynamic Desorption Elution on NKA-9 Resin Column

**Figure 9:**
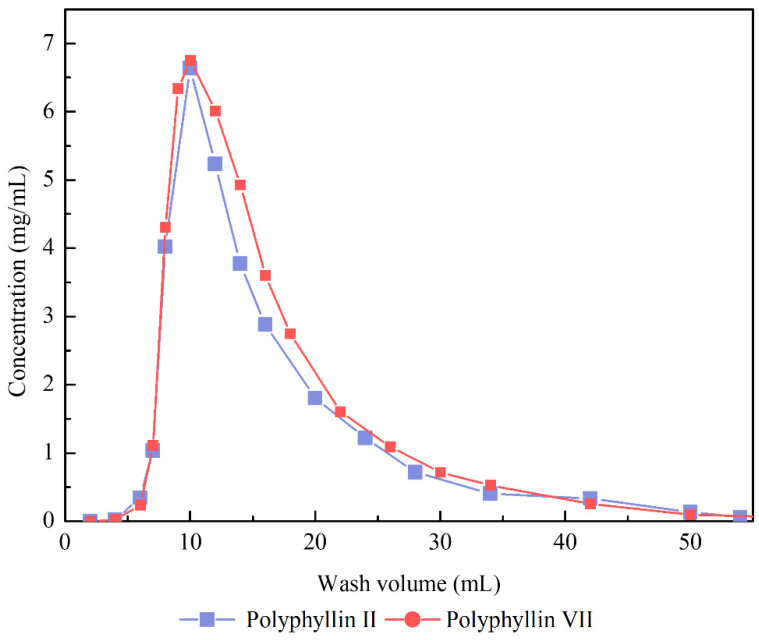
Isocratic elution curve of polyphyllin II and polyphyllin VII by 60% (*v*/*v*) ethanol.

The optimum elution volume and concentration were determined by isocratic elution and gradient elution, respectively. The elution curve was obtained by elution with 60% ethanol (Figure 9). The elution behaviors of polyphyllin II and polyphyllin VII showed a high degree of similarity. When the eluent volume was 10 mL, the eluent reached maximum concentration and was completely eluted at 55 mL for two saponins. Therefore, the amount of eluent could be 10 BV.

**Figure 10:**
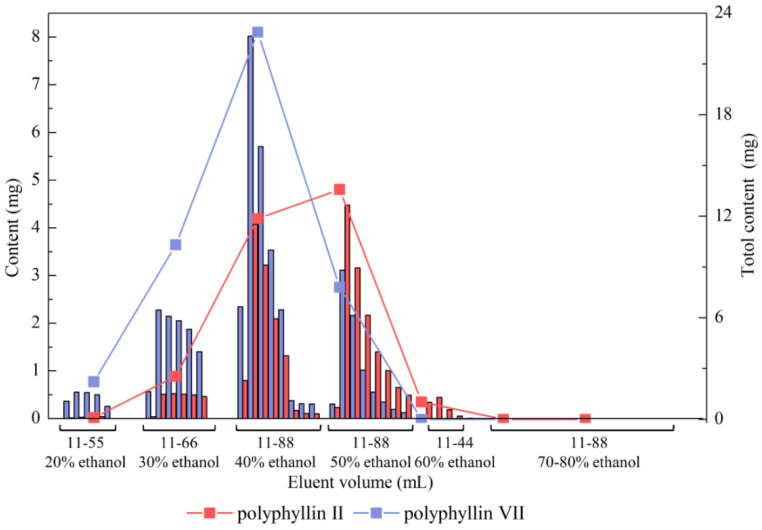
Effects of the concentration and volume of ethanol solution on NKA-9 column separation of two saponins.

The results of gradient elution are shown in Figure 10. The elution processes of polyphyllin II and polyphyllin VII were presumably the same, and desorption ability evidently changed together along with the ratio of ethanol in water. When a 20% ethanol solution was used, polyphyllin II and polyphyllin VII hardly flowed out. Although there was little polyphyllin II in the eluent of 20% ethanol, considerable impurities were desorbed at this time. The desorption capacity increased and reached a maximum when the ethanol concentration was 40%. During elution with 30% to 50% ethanol, most of the two saponins could be washed. Therefore, 10 BV 20% ethanol was used for impurity removal. Polyphyllin II and polyphyllin VII completely flowed out of the resin at approximately 44 BV 50% ethanol. The final purities, recovery rates, and yields of the two compounds are listed in Table 9.

Table 9 shows that the purities of polyphyllin II and polyphyllin VII in the obtained extract after treatment with NKA-9 resin were improved from 2.04% and 1.74% to 35.28% and 49.69%, corresponding to increases of up to 17.3 and 28.6 times, respectively. The recoveries of the two compounds were successfully recycled to 68.30% and 88.65% in the eluent of 30–50% ethanol. The final total elution yield of the two saponins reached 93.16%. In the other studies, Zhou et al. adopted the XAD-7HP resin column for a dynamic experiment; the purity of total steroidal saponins obtained by this method was, however, only 13.86% [50]. Wang et al. improved the purity of total flavonoids up to 50.57% with the help of purification by the HPD-600 resin, but the recovery rate was 76.4%. In our results, the purity of total saponins (polyphyllin II and polyphyllin VII) was 84.97%, and the recovery rate was 76.98% (Table 9), which is significantly higher than their purity levels.

**Figure 11:**
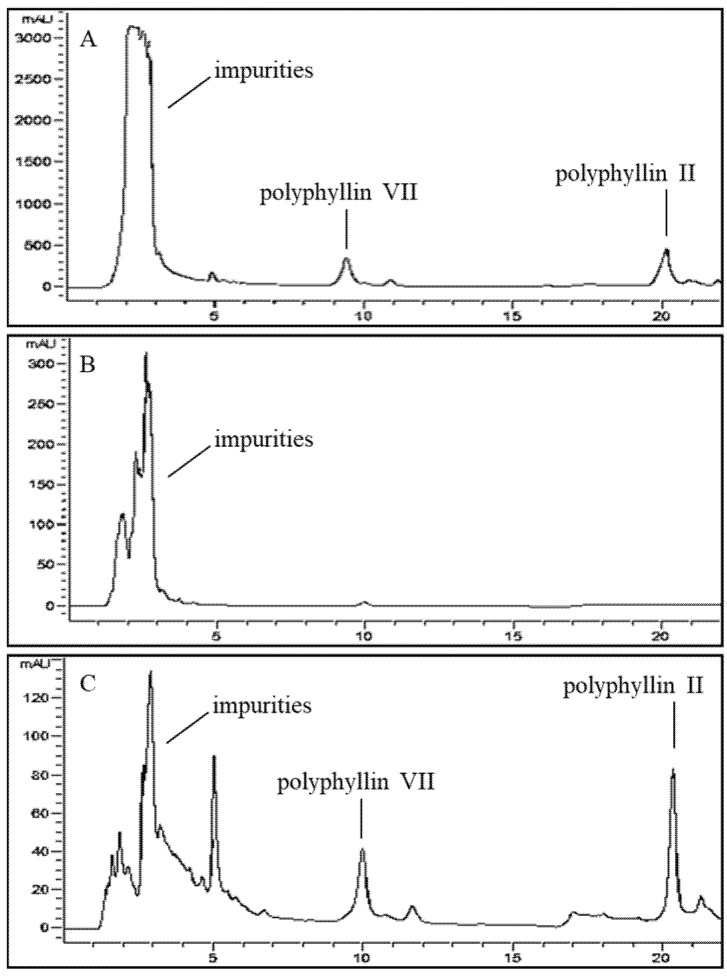
HPLC chromatograms of the samples: Sample solution before treatment (**A**); Samples eluted by 20% ethanol (**B**); Sample solution after treatment with the NKA-9 resin (**C**).

HPLC chromatograms of the sample solution before and after separation on the NKA-9 resin column are shown in Figure 10. Clearly, many impurities were eliminated after elution with 20% ethanol (Figure 11B). The relative contents of polyphyllin II and polyphyllin VII improved distinctly. The results clearly state that the optimized adsorption-desorption process of the NKA-9 resin had a good effect on the separation and purification of polyphyllin II and polyphyllin VII.

## 4. Conclusions

In this study, we developed an effective method to separate and purify polyphyllin II and polyphyllin VII from *P. polyphylla* var. *yunnanensis* extracts. The suitable NKA-9 resin was screened out by diverse data and model fitting results. The optimized conditions of the adsorption-desorption process were 35 °C, 10 BV, and 1 mL/min as the adsorption temperature, loading volumes, and flow rate, respectively; 10 BV 20% (*v*/*v*) ethanol as the impurity removal system; and 44 BV 50% (*v*/*v*) ethanol as the elution system in the gradient elution. With optimized conditions, the recoveries of polyphyllin II and polyphyllin VII were 68.30% and 84.61%, respectively, and purification multiples reached 17.3- and 28.6-fold, respectively. The efficiency of separation and purification of polyphyllin II and polyphyllin VII was greatly enhanced compared to conventional methods. This newly developed method is applicable and effective for large-scare industrial separation and purification of polyphyllin II and polyphyllin VII with lower cost, higher efficiency, and procedural simplicity.

## Figures and Tables

**Table 1:** Physical parameters and moisture contents of the macroporous resins.

Resin	Surface Area (m^2^/g)	Average Pore Diameter (nm)	Moisture Content (%)	Polarity
XAD7HP	≥380	4.5	70.65	Weak polar
HPD-100	650–700	8.5–9.0	69.00	Nonpolar
HPD-500	500–550	5.5–7.5	67.66	Polar
NKA-9	500–550	10.0–12.0	72.50	Polar
D101	480–520	25.0–28.0	67.50	Nonpolar
AB-8	480–520	13.0–14.0	71.64	Weak polarity
HP-20	500–600	29.0–30.0	67.00	Nonpolar

**Table 2:** The mobile phase consisted of A (water) and B (acetonitrile) using the gradient elution method.

Time (min)	A (%)	B (%)
0.0	57.0	43.0
13.0	57.0	43.0
14.0	45.0	55.0
22.0	45.0	55.0

**Table 3:** Summary of obtaining parameters from different models.

	Polyphyllin II				Polyphyllin VII		
	NKA-9	HPD-100	AB-8		NKA-9	HPD-100	AB-8
Pseudo-first-order model							
Q_e_	7.925	6.618	6.640		14.068	12.190	11.169
K_1_	0.083	0.108	0.135		0.145	0.184	0.133
R^2^	0.951	0.967	0.964		0.911	0.855	0.841
Pseudo-second-order model							
K_2_	0.015	0.051	0.032		0.039	0.026	0.010
R^2^	0.974	0.943	0.967		0.994	0.946	0.943
Weder-Morris intragranular diffusion model							
K_p_ *	1.220	1.082	1.212		1.858	0.835	1.107
Bangham model							
K	11.264	17.365	12.164		63.368	96.376	83.323
a	0.801	0.655	0.707		0.742	0.548	0.356

* K_p_ represents the reaction rate of the main adsorption.

**Table 4:** The comparison of coefficient in different studies.

Resin	Material	Coefficient of Determination (R^2^)		Reference
		Pseudo-First-Order Model	Pseudo-Second-Order Model	
HPD-300	three chromones (Prim-O-glucosylcimifugin, cimifugin, and 5-O-methylvisamminoside)	0.983, 0.935, 0.967	>0.990	[48]
NKA-II	total flavonoids (*Pteris ensiformis* Burm.)	0.974	0.998	[49]
XAD-7HP	total steroidal saponins (*Ophiopogon japonicus* (L. f.) Ker-Gawl)	0.973	0.990	[50]
NKA-9	two saponins (polyphyllin II and polyphyllin VII)	0.951, 0.911	0.974, 0.994	this study

**Table 5:** Correlation coefficients of the Langmuir and Freundlich equations.

Temperature (°C)	Langmuir Equation				Freundlich Equation		
	Q_m_	K_L_	R^2^		K_F_	1/n	R^2^
Polyphyllin II							
25	26.323	0.240	0.965		9.434	1.153	0.972
30	34.879	0.198	0.979		9.488	1.222	0.986
35	57.111	0.067	0.982		10.504	1.899	0.984
Polyphyllin VII							
25	23.064	0.777	0.973		6.925	2.240	0.994
30	10.209	0.584	0.981		4.047	2.058	0.992
35	38.722	0.799	0.985		14.305	1.835	0.982

**Table 6:** Thermodynamic parameters for the adsorption of two saponins on NKA-9.

	Polyphyllin II				Polyphyllin VII		
T (K)	ΔG° (kJ/mol)	ΔS° (kJ/mol)	ΔH° (kJ/mol)		ΔG° (kJ/mol)	ΔS° (kJ/mol)	ΔH° (kJ/mol)
298	−2148.80	46.17	8198.35		−1106.06	201.86	55360.13
308	−1348.45				−1395.48		

**Table 7:** Comparison of optimal temperature for different resins.

Resin	Material	Optimal Temperature	Reference
NKA-II (Polar)	total flavonoids (*Pteris ensiformis* Burm.)	25 °C	[49]
HPD-300 (nonpolar)	three chromones (Prim-O-glucosylcimifugin, cimifugin, and 5-O-methylvisamminoside)	25 °C	[48]
NKA-9 (Polar)	two saponins (polyphyllin II and polyphyllin VII)	35 °C	this study

**Table 8:** Two model fitting parameters of dynamic data on NKA-9.

Material	Pseudo-First-Order Model				Pseudo-Second-Order Model	
	Q_e_	k_1_	R^2^		k_2_	R^2^
Polyphyllin II	15.479	0.092	0.972		0.00391	0.861
Polyphyllin VII	13.132	0.116	0.977		0.00266	0.902

**Table 9:** Results of the optimization elution method for the two saponins.

Concentration of Ethanol (%)	Polyphyllin II				Polyphyllin VII				Total		
	P (%)	R (%)	Y (%)		P (%)	R (%)	Y (%)		P (%)	R (%)	Y (%)
20	0.24	0.24	─		5.36	4.56	─		5.6	2.54	─
30–50	35.28	68.30	68.46		49.69	84.61	88.65		84.97	76.98	93.16

P, Purity (%); R, Recovery (%); Y, yield (%).

## Data Availability

The data presented in this study are available in the article.
